# Long-Term Outcomes of One Anastomosis Gastric Bypass: A Systematic Review and Meta-Analysis of 5-Year and Beyond

**DOI:** 10.1007/s11695-025-08339-w

**Published:** 2025-10-15

**Authors:** Anuja Mitra, Amit Bhambri, Matyas Fehervari, Chetan Parmar

**Affiliations:** 1 Department of Surgery & Cancer, Imperial College London, London, United Kingdom; 2 Bhambri Bariatrics, Bhambri Hospital, Ludhiana, India; 3 Kent and Medway Medical School, University of Kent & University of Canterbury Christchruch, United Kingdom; 4 Tunbridge Wells Hospital, Royal Tunbridge Wells, United Kingdom; 5 Whittington Health NHS Trust, London, United Kingdom; 6 Department of Targeted Intervention, University College London, London, United Kingdom

**Keywords:** OAGB; long- term, Obesity associated diseases resolution, Weight loss, Revisional surgery

## Abstract

**Background:**

One-anastomosis gastric bypass (OAGB) has gained global prominence as the third most performed bariatric procedure. Despite evidence of short-term efficacy, long-term outcomes remain understudied.

**Methods:**

This PRISMA-compliant systematic review and meta-analysis evaluated long-term (≥ 5 years) outcomes of OAGB as primary and revisional procedures. Quality assessment and bias evaluation were conducted systematically.

**Results:**

Analysis included 32 studies with 19,125 patients (76% primary OAGB) from 14 countries with mean follow-up of 6.7 years. At five years, mean excess weight loss(EWL) was 75%, increasing to 77% beyond five years. Obesity associated diseases resolution was substantial: type 2 diabetes (80%), obstructive sleep apnea (89%), and hypertension (61%). Complications were minimal: bile reflux (4%), marginal ulceration (2%), and malnutrition (1%). For revisional OAGB, %EWL at five years was 71%. The conversion rate to other bariatric procedures (all RYGB) was reported in 3% of patients following OAGB.

**Conclusion:**

This analysis demonstrates OAGB's effectiveness for sustained weight loss and obesity associated diseases improvement at ≥ 5 years, supporting its role in long-term obesity management as a primary and revisional bariatric intervention.

**Supplementary Information:**

The online version contains supplementary material available at 10.1007/s11695-025-08339-w.

## Introduction

One Anastomosis Gastric Bypass (OAGB) is a well-established bariatric surgical procedure designed to facilitate weight loss and improve obesity-related comorbidities. First introduced by Rutledge in 1997 and subsequently refined by Carbajo and Caballero in 2002, OAGB has gained widespread acceptance and is now the third most commonly performed metabolic bariatric surgery (MBS) globally [1, 2]. Its increasing popularity is attributed to its technical simplicity, reduced operative time, and lower complication rates compared to Roux-en-Y Gastric Bypass (RYGB), whilst maintaining comparable—and in some studies superior—weight loss outcomes, particularly in patients with severe and complex obesity [3, 4]. As a testament to its efficacy and safety, OAGB has been formally endorsed as a primary MBS by both the International Federation for the Surgery of Obesity (IFSO) [5, 6], and the American Society for Metabolic and Bariatric Surgery (ASMBS) [7]. Despite its established role in MBS, there remains a paucity of information regarding its long-term outcomes.

The surgical technique of OAGB involves the creation of a long, narrow gastric pouch and a single gastrointestinal anastomosis [8]. While numerous studies have assessed perioperative and medium-term outcomes, concerns regarding the incidence of biliary reflux into the stomach and oesophagus, which may contribute to an increased risk of marginal ulcers and potential long-term malignancy [9–11]. To objectively assess these concerns, multiple studies have provided short- and mid-term data consistently demonstrating the efficacy of OAGB in achieving significant weight loss, resolving the metabolic syndrome, and improving obesity-related comorbidities, all while maintaining an acceptable safety profile [12, 13]. However, despite its increasing utilisation and integration into standard clinical practice, there remains a significant gap in knowledge regarding the long-term safety, durability, and complication profile of OAGB. Potential long-term risks, including an elevated incidence of gastroesophageal reflux, quality-of-life impairments, and malnutrition, remain speculative due to the absence of high-quality, longitudinal studies [14–16]. At present, no comprehensive evaluation exists on the long-term outcomes of OAGB beyond five years.

This study aims to address this critical gap by providing the first large-scale, global assessment of the long-term outcomes of OAGB, both as a primary and revisional procedure. By elucidating the extended safety, efficacy, and complication profile of OAGB, this research will contribute valuable insights to the bariatric surgery landscape and help identify patient subgroups that may derive the greatest benefit from this procedure.

## Methods

Following prospective registration on PROSPERO, a comprehensive literature search was performed in accordance with the recommendations of the preferred reporting items for systematic reviews and meta-analysis guidelines (PRISMA) to identify scientific publications reporting any weight related and clinical outcomes in adult patients who underwent OAGB as an index or revisional procedure. Searched databases included MEDLINE (1946 to 12 February 2025), EMBASE (1947 to 12 February 2025) via the Ovid platform and the Cochrane Review Library. Reference lists of eligible articles were also hand-searched for additional publications. The full search strategy can be found in supplementary file 1. All variations in the spelling, including truncated search terms using wild card characters and the “related articles” function, were used in combination with the Boolean operators AND OR.

## Selection of Studies: Inclusion and Exclusion Criteria

Following deduplication of search results, titles and abstracts were screened by two independent reviewers (A.T.M and A. B) with disagreements in study inclusion resolved by a third independent reviewer (C.P). The inclusion criteria required articles to report pre- and post- OAGB clinical outcomes and anthropometric parameters with follow up for 5 years or greater. Comparative cohort studies, non-randomised prospective studies, and randomised controlled trials published in the English language were included. Case series, case reports, narrative reviews, editorials, conference abstracts, studies with fewer than five participants or any study with follow up less than 5 years and were excluded.

## Data Extraction and Quality Assessment

Information extracted included year of publication, study design, sample size, country of study, length of follow up, baseline demographics of patients, index or revision OAGB procedure, length of bypassed biliopancreatic limb (BPL) (cm), pre- and post- operative anthropometric data [absolute weight (kg), body mass index (BMI), excess weight loss (%EWL), excess BMI loss (EMBIL), weight gain (kg)], and the following clinical outcomes; improvement or remission of type 2 diabetes mellitus (T2DM), obstructive sleep apnoea (OSA) and hypertension (HTN), evidence of biliary reflux, rate of marginal ulceration, malnutrition, iron (Fe) deficiency anaemia, need for revisional surgery and quality of life (QoL).

Case control and cohort studies were appraised for quality and rigorousness using the Newcastle–Ottawa Scale (NOS) [17] and randomised controlled trials (RCTs) were appraised using the JADAD score [18].

## Outcome Measures

All data was extracted up to the longest follow up. Data was pooled for effect estimates for meta-analysis if there were 2 or more studies. Baseline characteristics were reported as mean values with standard deviations and percentages. Modulation in outcomes between pre- and post- surgery groups were reported as standardised or weighted mean differences, or percentages with 95% confidence intervals.

## Statistical Methods

Data analysis was performed using STATA statistics Software, Version 16.0. StataCorp LCC, TX. Weighted means of single values were calculated using random effect analysis with the DerSimonian and Laird method [19]. For sub-group analysis based on limb length, groups were dichotomised into ‘standard’ (< 200 cm) or ‘long (≥ 200 cm) BPL. Descriptive statistics were described as mean and standard error of the mean. Data for the meta-analysis was analysed using a random effects model and statistical heterogeneity was calculated using *I*^*2*^. An *I*^*2*^ of < 30% was considered as low, 30–60% as moderate and > 60% as high heterogeneity. Results were computed and represented on forest plots with meta-analyses.

## Results

### Summary of Results

**Fig. 1:**
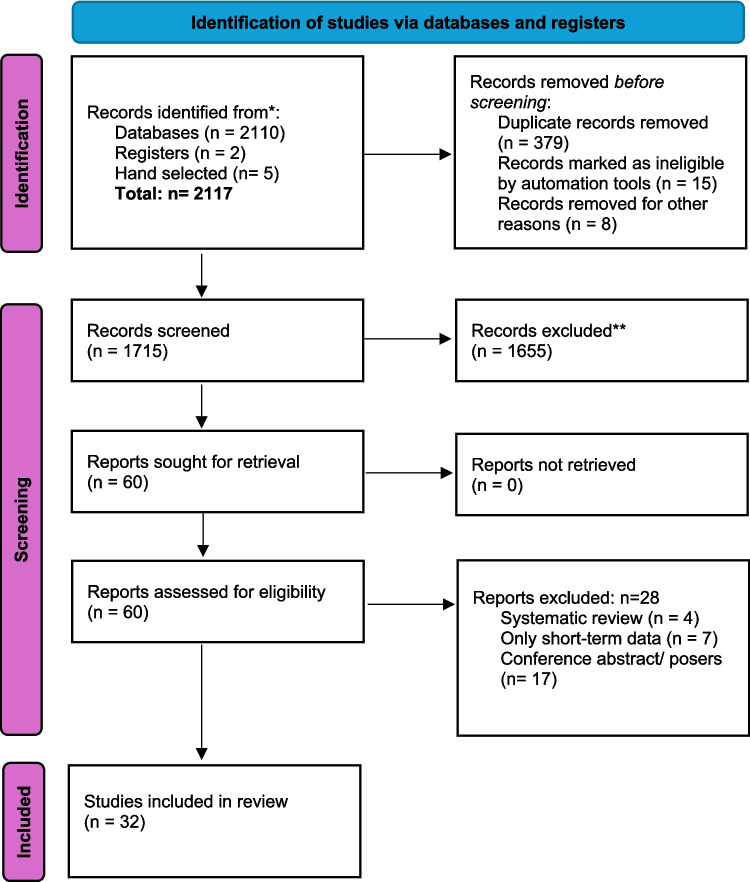
PRISMA flowchart

Our review of the literature identified 32 studies across 14 countries which met the inclusion criteria for the final meta-analysis (see PRISMA flowchart, Fig. 1). Among these, 8 studies were a combination of prospective cohort and case control studies, 22 were retrospective and 3 were RCTs. The total number of participants was 19,125. The baseline mean BMI was 44.8 ± 4.9 kg/m^2^, average age was 40.8 ± 6.4 years and 68% were female. Twenty- six studies evaluated OAGB as a primary procedure, 4 as revisional procedure and 3 included both groups. The mean follow-up period was 6.7 years, with the longest follow-up extending to 20 years [20].

### Quality Assessment

NOS reporting standards of the studies ranged from 5 to 9, with the median score being 7 and the median JADDAD score being 4, rating the included studies as high quality. Study heterogeneity as reflected by the *I*^*2*^ statistic ranged from 0- 100%. Study characteristics, patient demographic data and quality assessment scores can be found in Table 1.

**Table 1:** Study characteristics, patient demographics and quality assessment of included studies

Study details			Baseline Patient demographics					Study			
Author	Publication year	Country	Study design	Sample size (n)	Mean BMI (kg/m^2^)	Mean age (years)	Gender (% female)	OAGB (primary or revisional)	Length of F/U (years)	NOS/JADDAD* score (RCTs)	BP limb length (< 200 cm or ≥ 200 cm)
Rutledge [21]	2005	USA	Prospective case series	2410	46 ± 7 (34–74)	39 (14–78)	85	primary	6	6	180
Lee A [22]	2008	China	Prospective case series	820	39.3 ± 8	30.7 ± 8.6	75	primary	5	7	(100 cm for BMI < 35, 200 cm for BMI 35–50, 300 cm for BMI > 50
Lee B [23]	2012	China	Retrospective cohort study	1163	41.1 ± 6.1	31.6 ± 9.1	73	primary	9	8	200
Noun [24]	2012	Lebanon	Retrospective study of a prospectively maintained database	1000	42.5 ± 6.3	33.2 ± 10.2	66	primary and revision	6	7	150 cm (and increased by 10 cm for each BMI point above 40)
Clarke [25]	2013	New Zealand	Retrospective case series	156	46 (35–64)	44 (18–63)	78	primary	5	6	150–200
Musella A [26]	2013	Italy	Prospective case series	974	48 ± 4.6	39.4	51	primary	6	7	200
Kular A [27]	2014	India	Retrospective review of prospectively maintained data	1054	43.2	38.4	68	primary	6	8	200
Chevallier A [28]	2015	France	Prospective cohort study	823	46.7 ± 9	40.6 ± 17.9	71	primary	7	6	200
Chevallier B				177	44.5 ± 6.4	43.6 ± 10.5	79	revision	5	6	200
Kular B [27]	2015	India	Retrospective review of prospectively maintained data	128	33.4 ± 5	41.6 ± 10.2	64	primary	7	6	150
Peraglie [29]	2015	USA	Prospective case series	758	43 (33–61)	64 (60–74)	62	primary	6	8	180
Bruzzi [30]	2015	France	Retrospective case series	126	47 ± 8	50 ± 10	79	primary	5	8	200
Carbajo [31]	2016	Spain	Retrospective review	1200	46 ± 13.3	43 (12–74)	62	primary	6	8	> 200 (> 200–350)
Jammu [32]	2016	India	Retrospective cohort study	167	56.5 (40–73)	46.5	71	primary	7	8	200
Alkhalifah [33]	2017	Singapore	Retrospective analysis, of a prospective bariatric database	1731	40.4 ± 7.7	33.8 ± 10.4	70	primary	15	7	150- 200
Musella B [34]	2017	Italy	Retrospective review of prospectively maintained data	2678 primary, 427 revision	45.4 ± 3.6	42.2 ± 3.8	70	primary and revision	5	8	217 ± 13.8 cm (165–260-cm range)
Taha [35]	2017	Egypt	Prospective case series	1520	46.8 ± 6.6	37.2	63	primary	6	7	150–300
Almalki [36]	2018	Taiwan	Retrospective cohort study	81	37.2 ± 9.2	35.7 ± 9.8	74	revision	5	7	200
Bhandari [37]	2019	India	Retrospective cohort study	57	42	46.4	33	primary	5	7	250
Neuberg [38]	2019	France	Retrospective study	163	41.2 ± 6.5	41 ± 11.4	66	primary and revision	8	8	150
Poghosyan [39]	2019	France	Retrospective analysis of a prospectively collected database	72	43.6 ± 7	47 ± 10 (25–63)	72	revision	5	7	200 cm in 38 patients (52.7%), 150 cm in 34 patients (47.3%)
Baig [40]	2019	India	Retrospective cohort study	203	45.1 ± 8.82	43.1 ± 11.4	54	primary	5	8	150–210
Level [41]	2020	Venezuela	RCT	9	42.9 ± 5.5	37.5 ± 6.6	100	primary	5	4*	200
Liagre [42]	2020	France	Retrospective review of prospectively maintained data	115	43.2 ± 5.8	37.2 ± 11.7	94	primary	8	7	150
Miller [43]	2020	Austria	Prospective case series	12	57.5 ± 6.3	38.2 ± 6.5	42	primary	5	6	200
Almuhanna [20]	2021	Taiwan	Retrospective analysis was conducted of a prospective bariatric database	2223	40.3 ± 11.9	35.4 ± 11.4	70	primary	20	9	200, 150 for BMI < 35, then 10 cm increase for each BMI > 35
Carandina [44]	2021	France	Retrospectively reviewed of prospectively collected data	385	44.3 ± 6.7	43.2 ± 9.7	89	primary	10	8	200
Jain [45]	2021	India	RCT	73	45.3 ± 8.24	42.8 ± 10.4	58	primary	5	4*	150–180
Soong [46]	2021	Taiwan	Retrospective cohort study	246	56.2 ± 5.8	31.9 ± 9.7	45	primary	5	8	250–350
Makkapati [47]	2024	India	Retrospective observational study	126	48 ± 20	41 ± 12.5	25	primary	10	7	180- 200 cm for BMI 36–40, if > 40, 250 cm BPL limb
Robert [48]	2024	France	RCT	114	44 ± 5.6	43 ± 10.8	75	primary	5	3*	200
Shahmiri [49]	2024	Iran	Retrospective observational study	53	45.1 ± 6.7	39.4 ± 11.9	82	revision	5	7	150–200
Van der Laan [50]	2024	Netherlands	Prospective cohort study	860	43 (40- 46)	47(38–53)	75	primary	5	9	150–180

## Long Term OAGB Data

### Weight Loss

**Fig. 2:**
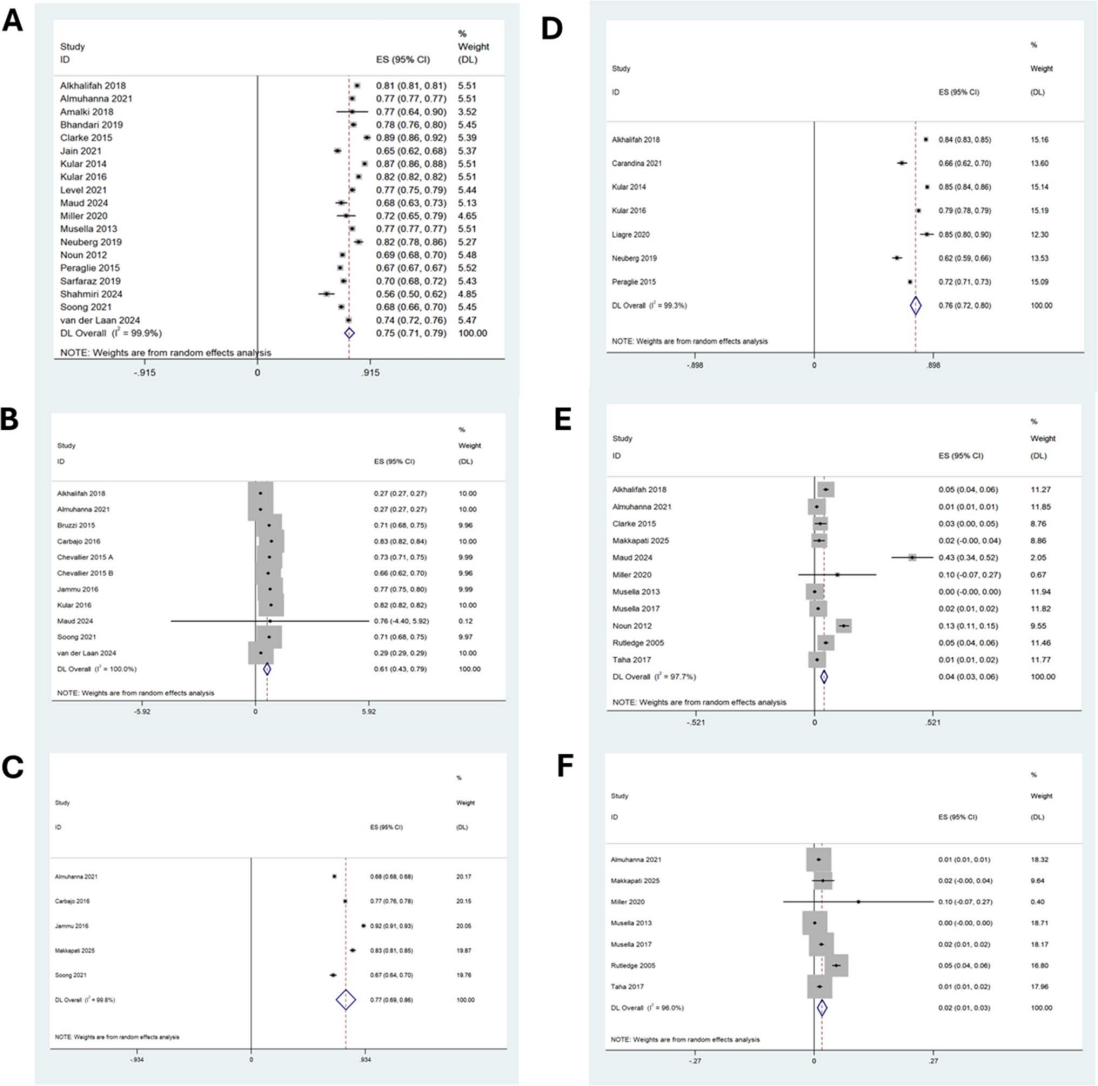
Forest plots illustrating random effects modelling for weighted mean differences in weight outcomes following OAGB **a**) %EWL at 5 years, **b**) EMBIL at 5 years, **c**) %EWL at 5 years with biliopancreatic limb (> 200 cm), **d**) %EWL at 5 years with standard (< 200 cm) biliopancreatic limb, **e**) % weight recurrence at 5 years and **f**) % weight recurrence with biliopancreatic limb (> 200 cm)

At five years post-OAGB, the mean absolute weight loss was 117 kg (95% CI: 106.5–128.1, I^2^ = 99.8%) across nine studies (n = 8,150), corresponding to a 75% EWL (95% CI: 71–79, I^2^ = 99.9%) in 11,296 patients, Fig. 2a. Beyond five years, the mean %EWL remained at 77% (95% CI: 72–81, I^2^ = 99.8%, n = 9,752). Eleven studies (n = 9,490) reported EMBIL, demonstrating a 61% reduction at five years (95% CI: 43–79, I^2^ = 100%), Fig. 2b.

A BPL length ≥ 200 cm (n = 3,635) was associated with a %EWL of 76% at five years (95% CI: 74–77, I^2^ = 93.4%) and 77% beyond five years (95% CI: 69–86, I^2^ = 99.8%, n = 4,268), Fig. 2c. In comparison, a BPL length < 200 cm resulted in marginally lower %EWL, with 74% at five years (95% CI: 68–80, I^2^ = 99.9%, n = 7,661) and 76% beyond five years (95% CI: 72–80, I^2^ = 99.3%, n = 5,484), Fig. 2d.

### Weight Recurrence Following OAGB

Eleven studies using data from 12,944 patients determined the long- term recurrent weight gain (RWG) following OAGB as an index procedure. RWG was reported in 4% of patients overall [95% CI 3; 6, I^2^ 97.7%], Fig. 2e. When limb length was ≥ 200 cm, weight gain was 2% [95% CI 1; 3, I^2^ 96%], (n = 9943) Fig. 2f, in comparison to 8% [95% CI 5; 12, I^2^ 97.9%] in patients with a standard (< 200 cm) BPL length (n = 6931), supplementary Fig. 2a.

### Type 2 Diabetes Mellitus

**Fig. 3:**
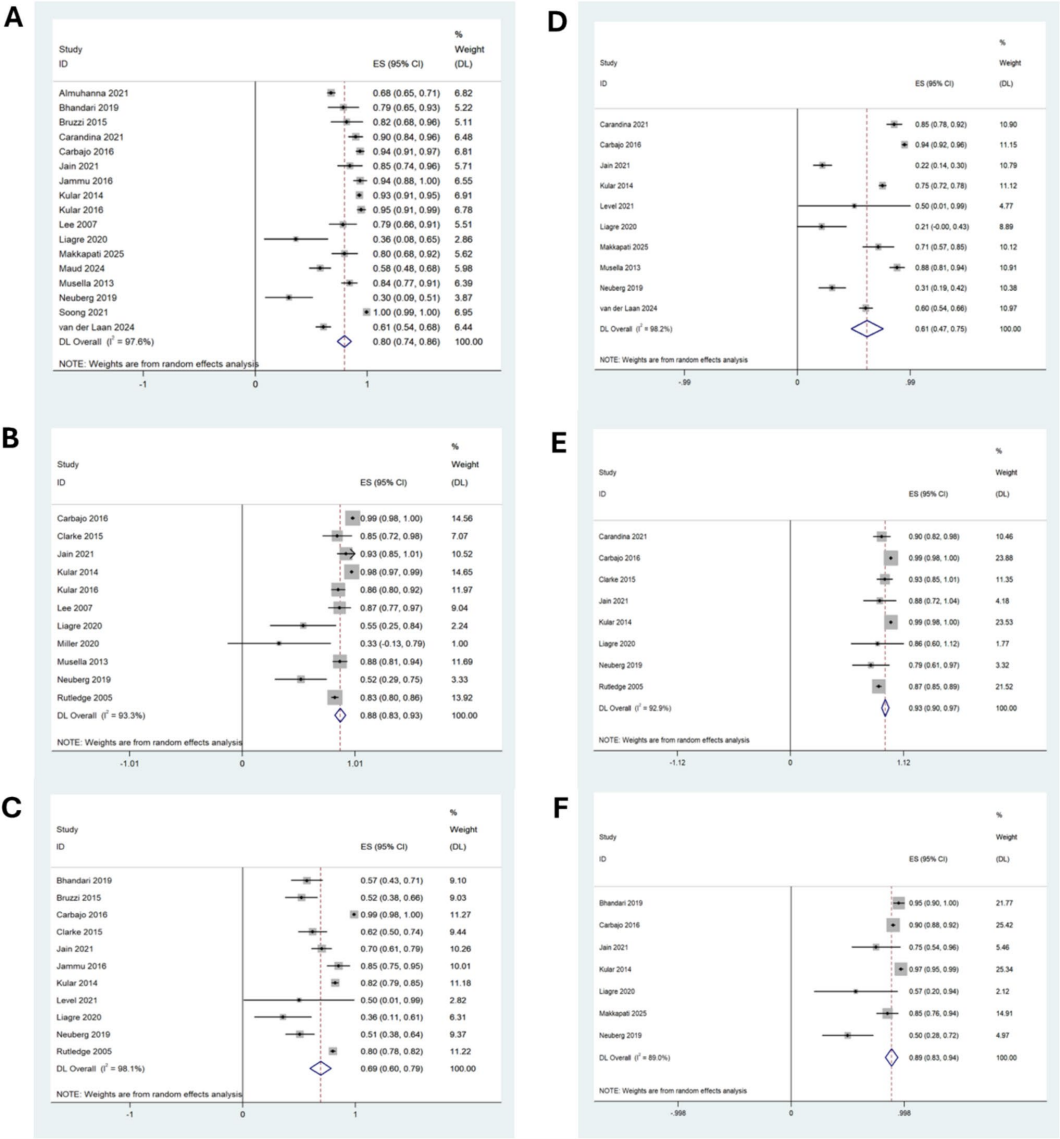
Forest plots illustrating random effects modelling for weighted mean differences in obesity related comorbidities following long term OAGB **a**) % type 2 diabetes mellitus (T2DM) resolution, **b**) % T2DM improvement, **c**) % hypertension (HTN) improvement, **d**) % HTN resolution, **e**) % Obstructive Sleep Apnoea (OSA) improvement and **f**) % OSA resolution

Long-term complete resolution of T2DM was observed in 80% of patients (95% CI: 74–86, I^2^ = 98%, n = 3,226) following OAGB, with overall T2DM improvement in 88% (95% CI: 83–93, I^2^ = 93%, n = 1,809; 11 studies), Fig. 3a, b respectively.

Subgroup analysis by BPL length demonstrated superior metabolic outcomes with a longer limb (≥ 200 cm), achieving complete T2DM resolution in 85% (95% CI: 75–95, I^2^ = 98%) and improvement in 91% (95% CI: 85–96, I^2^ = 96%, n = 1,583), supplementary Fig. 2b. In contrast, a BPL < 200 cm, based on data from eight studies (n = 1,262), was associated with lower T2DM resolution rates of 73% (95% CI: 62–83, I^2^ = 96%), supplementary Fig. 2c and T2DM improvement in 84% (95% CI: 75–93, I^2^ = 95%, n = 1,478).

### Hypertension

With random effects modelling, 11 studies with a total of 2827 patients demonstrated improvement in HTN in 69% of patients following OAGB [95% CI 60; 79, I^2^ 98%] and seven studies with 1419 patients demonstrated HTN resolution in 61% of patients [95% CI 47; 75, I^2^ 98%], Fig. 3c, d respectively.

### Obstructive Sleep Apnoea

Eight studies with a total of 2367 patients demonstrated OSA improvement following OAGB in 93% [95% CI 90; 97, I^2^ 93%] and OSA resolution in 89% of patients [95% CI 83; 94, I^2^ 89%] (n = 1709), Fig. 3e, f.

### Bile Reflux

**Fig. 4:**
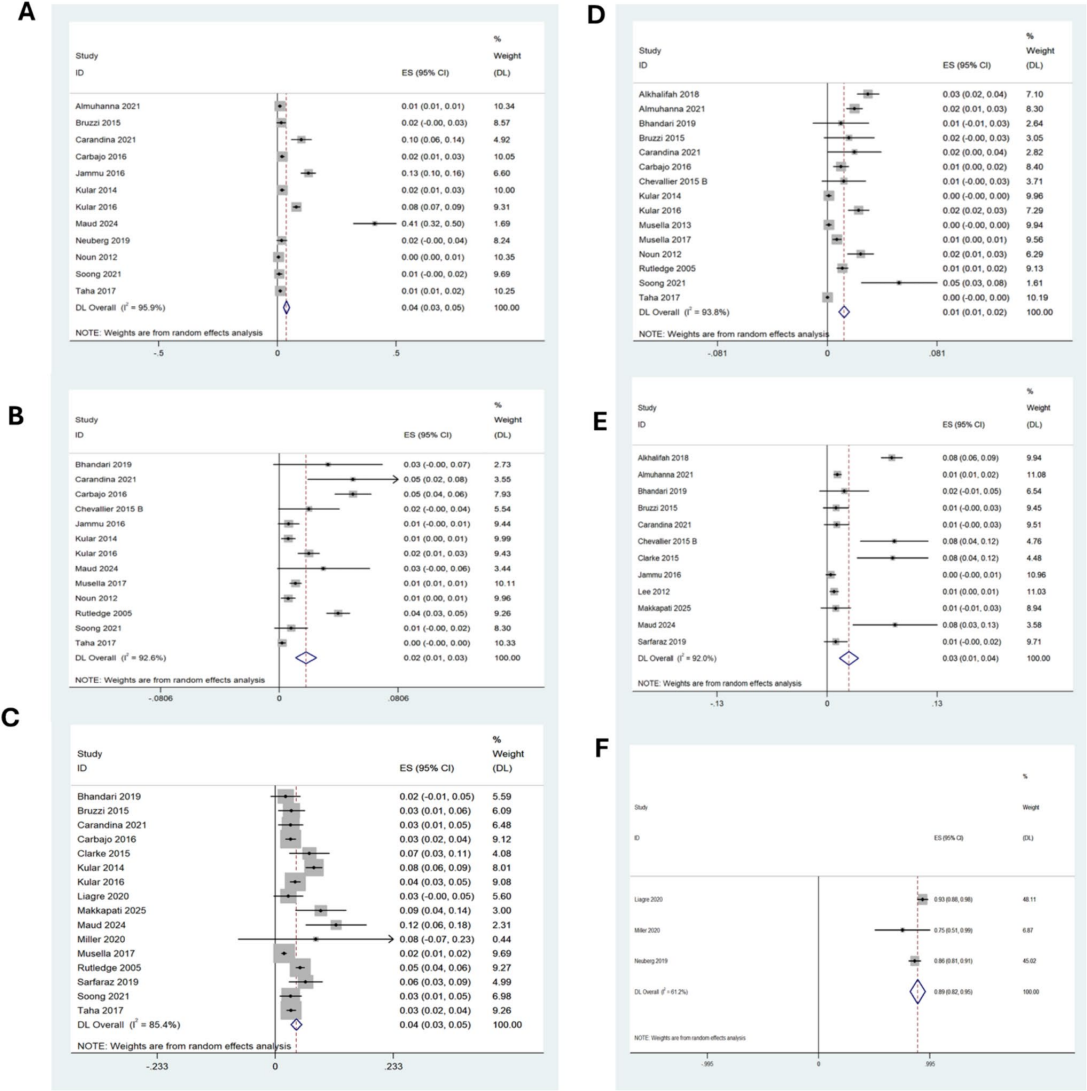
Forest plots illustrating random effects modelling for weighted mean differences long term OAGB outcomes for **a**) % de novo bile reflux, **b**) % marginal ulcers, **c**) % iron deficiency anaemia, **d**) % vitamin & mineral malnutrition, **e**) % conversion surgery rates to RYGB and f) quality of life scores

De novo bile reflux was reported in 4% of patients following OAGB (95% CI: 3–5, I^2^ = 96%, n = 9,831), Fig. 4a. Subgroup analysis by BPL length showed a lower incidence with a longer limb (≥ 200 cm), where 2% of patients (95% CI: 1–3, I^2^ = 91%, n = 6,891; 7 studies) developed bile reflux, in contrast, a shorter BPL length was associated with an risk of bile reflux at 5% (95% CI: 3–7, I^2^ = 97%, n = 5,514), supplementary Figs. 2d and e, respectively.

### Marginal Ulcers

Thirteen studies with data from 12,625 patients reported de novo marginal ulcers in 2% of patients [95% CI 1; 3, I^2^ 100%] following OAGB at 5 years, Fig. 4b.

### Anaemia

Sixteen studies comprising 11,762 patients assessed the incidence of iron deficiency anaemia following OAGB. Using a random-effects model, the overall prevalence of significant anaemia was 4% (95% CI: 3–5, I^2^ = 85.4%), Fig. 4c. The risk with a standard BPL limb length was 5% (95% CI: 4–6, I^2^ = 79%, n = 1,942) compared to 4% (95% CI: 3–5, I^2^ = 89.3%, n = 9,511) in those with a longer BPL length, supplementary Figs. 2f and g, respectively.

### Mineral Malnutrition

Long-term malnutrition, was reported in 1% of patients following OAGB (95% CI: 1–2, I^2^ = 100%, n = 17,141), Fig. 4d. Subgroup analysis by BPL length showed a similar prevalence, with malnutrition occurring in 1% of patients with a BPL ≥ 200 cm (95% CI: 0–1, I^2^ = 91.4%, n = 12,747; 12 studies) and in 1% of those with a standard limb length (95% CI: 1–2, I^2^ = 0%, n = 9,378), supplementary Figs. 2h and i, respectively.

### Conversion Surgery

The conversion rate to other bariatric procedures (all RYGB) was reported in 3% of patients following OAGB [95% CI 1; 4, I^2^ 92%] in 12 papers with 6826 patients, Fig. 4e.

### Quality of Life

Three studies with a total of 290 patients evaluated QoL using either the BAROS score or the Moorehead-Ardelt II (MA-II) questionnaire. Outcomes demonstrated an improvement in reported QoL in 89% [95% CI 82; 95, I^2^ 61%] of patients following OAGB in the long- term, Fig. 4f.

### OAGB as Revisional Procedure Data

**Fig. 5:**
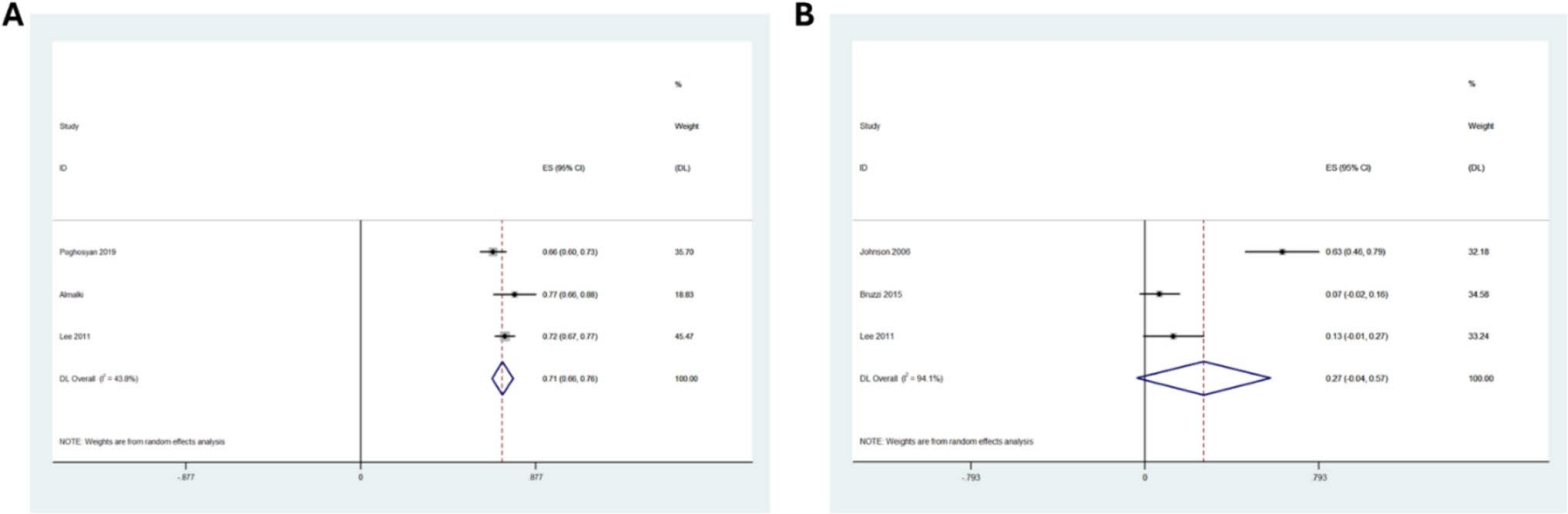
Forest plots illustrating random effects modelling for weighted mean differences for OAGB as a revisional procedure in terms of **a**) %EWL and **b**) % of bile reflux in revisional OAGB patients at 5 years

OAGB was performed as a revisional procedure in three studies comprising of 211 patients, all of whom had undergone primary restrictive bariatric procedures. At mean follow up of five years, the %EWL following revisional OAGB was 71% (95% CI: 66–76, I^2^ = 44%), Fig. 5a. However, data from three studies indicated that 27% (n = 23) of 85 patients (95% CI: −4 to 57, I^2^ = 94%) required further revision due to intractable bile reflux, though this finding was not statistically significant, Fig. 5b.

## Discussion

This study represents the first comprehensive, large-scale, single-arm analysis evaluating the long-term outcomes of OAGB as both a primary and revisional MBS. Seminal findings confirm that OAGB leads to significant and sustained weight loss over time, alongside substantial improvements—and in many cases, resolution—of obesity-related metabolic comorbidities. While long-term complications such as mineral malabsorption, RWG, bile reflux, and marginal ulcer formation were observed, their incidence did not exceed known expectations and were comparable to other endorsed bariatric procedures. Furthermore, OAGB was shown to be an effective revisional procedure, particularly for sustained weight loss, reinforcing its role as a safe and highly efficacious intervention in obesity management.

## Long-Term Weight Loss Outcomes

A key finding of this review is the substantial and sustained weight loss achieved with OAGB. At five years postoperatively, the mean %EWL was 76%, notably higher than the 50–60% typically reported for RYGB, underscoring the long-term effectiveness of OAGB as a primary weight-loss procedure [51, 52]. These results align with those of the multicentre RCT YOMEGA (NCT02139813) trial, which demonstrated the non-inferiority of OAGB compared to RYGB at two and five years [48, 53]. Results demonstrated a mean EMBIL of −87.9% for OAGB versus −85.8% for RYGB at 2 years, which was maintained at 5 years with a mean EMBIL of −75.6% for OAGB versus −71.4% for RYGB. Our findings mirror the long- term results over a longer follow-up period, reaffirming the durability of weight loss with OAGB.

RWG remains a concern across all bariatric procedures, though our review found it to be relatively low following OAGB (4% at five years), significantly lower than rates reported for sleeve gastrectomy (SG) (17.5%), RYGB (16.7%), and other OAGB studies (11.6%) [54]. While superior weight loss outcomes are achieved with OAGB in the short and long term, contributing factors for long-term RWG may include a combination of lifestyle habits, gastric dilation over time, and underlying comorbidities, highlighting the importance of careful patient selection, sustained motivation, and proactive management of dietary and psychological factors to optimise surgical success [55]. Optimising surgical outcomes necessitates careful patient selection, sustained motivation, and proactive management of dietary and psychological factors. Interventions for RWG include lifestyle modifications, pharmacotherapy, and in select cases, revision surgery with 3D modelling pre- operatively to enhance patient engagement [56]. Notably, conversion from OAGB to RYGB has been associated with weight gain, as demonstrated by Sargysan et al., who reported a mean BMI increase of 0.61 kg/m^2^ in 136 patients in the medium term. However, in cases where bile reflux necessitates conversion, the modest weight gain may be an acceptable trade-off for symptom relief [57].

## OAGB as a Revisional Procedure

OAGB has demonstrated efficacy as a revisional procedure, particularly in patients who previously underwent restrictive bariatric surgeries such as gastric banding or SG [3, 11, 58]. The primary indications for revision were weight loss failure, weight recurrence, or the recurrence of obesity-related diseases. Our findings indicate that long-term mean %EWL following revisional OAGB was 71%, consistent with previous literature. Kermansaravi et al. confirmed the effectiveness of OAGB as a revisional procedure following SG, showing sustained weight loss at five years [59]. However, while some studies report diminishing effects beyond two years, our analysis suggests a continued positive trajectory beyond five years.

When comparing revisional OAGB to RYGB, surgical selection should be tailored to the underlying indication. A meta-analysis by Santoro et al. involving 802 patients found that RYGB was superior for managing bile reflux, whereas OAGB was more effective for inadequate weight loss [60]. In our study, 27% of patients(n = 23/85) who underwent revisional OAGB required further revision due to intractable bile reflux. These findings support the conclusion that OAGB can be considered a safe and effective revisional procedure for weight loss failure but may need careful patient selection when bile reflux is the primary indication for revision. This should be preferably performed by experts in high volume centres [13, 31, 34]. More evidence including RCTs are required to study this comparison in future.

## Metabolic and Comorbidity Resolution

MBS has consistently proven to be highly effective in managing obesity-related medical comorbidities. In this review, we observed significant long-term benefits of OAGB, particularly in the improvement of HTN, T2DM, and OSA. A superiority of OAGB over RYGB is postulated to be its superior glycaemic control and higher remission rates for T2DM [61, 62]. Although the exact mechanisms underlying these benefits remain incompletely understood, they are thought to involve a complex interplay of weight-dependent and weight-independent factors, including hormonal regulation, the autonomic nervous system, bile acids, and the gut microbiota, along with their interrelationships. These mechanisms are the subject of ongoing investigation, in the ORDER trial (Efficacy and Safety of One Anastomosis Gastric Bypass versus Roux-en-Y Gastric Bypass for Type 2 Diabetes Remission) [63]. This multicentre, randomised controlled, open-label superiority trial specifically aims to evaluate the efficacy of OAGB over RYGB in achieving T2DM remission.

## Long-Term Safety and Complications

MBS is associated with certain risks, but when comparing OAGB to RYGB or SG, the long-term complications appear to be comparable. This is particularly commendable considering as the OAGB population often has higher rates of functional impairment and disease complexity, as measured by the obesity surgery-metabolic risk score [64]. OAGB and RYGB procedures carry the risk of anastomotic leaks [65, 66], vitamin and mineral deficiencies, including iron deficiency anaemia. Reported rates of iron deficiency anaemia following RYGB are approximately 16.7% at 10 years, whereas SG has a reported rate of 1.6% [67, 68]. These risks underscore the necessity for lifelong post-operative vitamin and mineral supplementation as the negative sequalae of iron deficiency anaemia may include hospitalisation in 54% of cases carrying an increased mortality rate of 18% [69]. In this review, we found that 4% of patients developed iron deficiency anaemia beyond five years after OAGB, a rate considered lower than RYGB, however it is difficult to corelate this with compliance of post-surgery supplementations. This has echoed the results of other large-scale studies. The GENEVA international cohort of 6770 patients undergoing MBS reported that RYGB had higher rates of perioperative complications compared to SG and OAGB [70]. Additionally, propensity-matched analysis from the Metabolic and Bariatric Surgery Accreditation and Quality Improvement Program (MBSAQIP) demonstrated that OAGB had a lower overall complication rate than RYGB, with no statistically significant difference when compared to SG [71].

In our study, mineral malnutrition was reported in 1% of cases, though this may be an underestimation due to variability in post-operative follow-up protocols across institutions. These findings are consistent with large-scale data, such as the study by Bandlamudi et al., which examined malnutrition in nearly 50,000 OAGB patients and reported an in-hospital treatment rate of 0.9% [72]. While longer BPL lengths have been associated with an increased risk of malnutrition, our analysis did not demonstrate a significant difference based on BPL, a result supported by existing literature. Gentileschi et al. similarly found no significant difference in malnutrition rates between OAGB and RYGB at six months postoperatively, reinforcing the notion that OAGB is not inferior to RYGB in terms of early nutritional outcomes [73]. These findings underscore the critical importance of structured, long-term follow-up to identify and mitigate potential nutritional deficiencies, ensuring optimal patient outcomes.

A point for discussion with OAGB is the incidence of de novo bile reflux, which in the short or long term can necessitate conversion to an alternative procedure. Long-term, bile reflux may also elevate the risk of gastroesophageal malignancies due to bile acid contact with the oesophageal lining [14, 48, 74]. This review found a 4% overall incidence of de novo bile reflux, with a higher risk (5%) in patients with shorter BPL lengths. This can be explained by the theory that when the BPL is longer the biliary-pancreatic enzymes have more chances of re-absorption in the long BPL and they reach the anastomosis in more deactivated form [2, 31]. Incidence rates in the literature vary widely, ranging from 7.8% to 55.5% [75, 76]. Factors such as shorter gastric pouch, inadequate repair of hiatus hernia and gastrointestinal anastomotic techniques can contribute to the incidence of bile reflux [8, 31, 34]. While OAGB has been shown to be non-inferior to RYGB for weight loss, the relatively high incidence of gastroesophageal reflux disease (GERD) raises concerns about its long-term effects. Felsenreich et al. objectively demonstrated a decrease in acid reflux but an increase in non-acid reflux after OAGB in the mid- term using gastroscopy, impedance studies and pH manometry. Results demonstrated chronic reflux exposure in the anastomosis, pouch, and distal oesophagus, even in asymptomatic patients, highlighting the need for routine follow-up to assess malignancy risk [77]. In contrast, Kermansaravi et al. found that 82% of patients experienced GERD improvement or remission after OAGB, similar to rates reported by Carbajo et al. [31], adding complexity to the ongoing debate about bile reflux in this procedure [59].

In this review, 2% of patients developed marginal ulcers long term, with some studies reporting rates as high as 16% [78]. The conversion rate to other bariatric procedures following OAGB was 3%, with all cases resulting in conversion to RYGB for intractable bile reflux. Given the available data, it may be advisable to carefully consider the choice of surgical procedure when counselling patients, particularly those with specific indications for surgery. In patients at high risk for bile reflux, OAGB should be avoided in favour of RYGB.

## Quality of Life and Surgical Optimisation

QoL improvements were reported in 89% of patients undergoing OAGB, highlighting the significant positive impact of the procedure on both physical and psychological well-being. However, a limitation of included studies is the lack of uniformity in the QoL assessment tools used. Two studies employed the BAROS score, which evaluates factors such as excess weight loss, improvement in comorbidities, and complications to provide a comprehensive assessment of MBS outcomes [79]. The Moorehead-Ardelt II (MA-II) questionnaire evaluates the psychosocial aspects of weight loss surgery, including patient satisfaction and emotional well-being [80]. While these are both recognised and validated post-operative QoL scales, the variation in tools used across studies may have impacted the validity and comparability of results.

Subgroup analysis in this study examined the impact of varying BPL lengths on outcomes following OAGB. There is accepted variability in BPL chosen by bariatric surgeons, with some opting for fixed lengths ranging from 150 to 200 cm, while others tailoring the BPL based on individual patient factors such as BMI, age, sex, diet, and comorbidities. A report on OAGB in the UK demonstrated the trend for change in BPL length has evolved over time, with a reduced use of 200 cm length and greater use of 150 cm BPL lengths [81]. It is common practice for patients with higher BMI to receive longer BPL lengths to enhance weight loss outcomes. Our findings demonstrate that a longer BPL (> 200 cm) was associated with greater weight loss, though not statistically significant, as well as more favourable improvements in obesity-related comorbidities. However, it is strongly advised that total small bowel length should be measured when longer BPL are considered for safe outcomes [31, 82]. Studies by Carbajo et al. have shown that OAGB obtained significantly greater weight loss and remission of dyslipidemia than the other techniques. And there was a trend towards greater T2D and hypertension remission rate after OAGB [82]. Furthermore, longer BPLs were correlated with a lower incidence of bile reflux. These findings are noteworthy, as previous literature has reported that bypassed limbs exceeding 200 cm deliver better weight loss outcomes, albeit potentially at the expense of more severe nutritional deficiencies [83]. This relationship warrants further investigation to establish optimal limb lengths that balances maximal weight loss efficacy with minimal nutritional complications.

A main strength of this review lies in the large-scale nature of the study for overall and subgroup analysis. The large sample size strengthens the validity and reliability of the study findings, enhancing its significance and strengthening interpretations. The long- term nature of the data offers novel and unique insights into key outcomes following OAGB over years so patients and clinicians alike can be informed about choices based on scientific evidence. The limitations of this paper include the heterogeneity in pooling large-scale studies as reflected in the range of *I*^*2*^ scores. We included the study by Miller et al. [43], which evaluated long-term outcomes following banded OAGB in patients with super-obesity, due to its unique focus on a niche procedure with limited long-term data. Given the scarcity of studies reporting five-year outcomes for this specific technique, we felt that excluding this study would omit valuable insights that could inform clinical practice. Looking ahead, long-term follow-up is crucial, especially in assessing the risk of reflux and its long-term consequences like oesophageal cancer, a major concern post-OAGB, however current existing published literature shows this not to be of any concern.

## Implications for Clinical Practice

OAGB has been safely implemented in the global bariatric surgery landscape and recognised as a mainstream bariatric procedure. Results from this analysis confirm the long-term safety and effectiveness of OAGB. Supported by published Delphi consensus guidelines, these findings identify the appropriate subgroups of patients for OAGB, with elderly individuals and those with severe or complex obesity and associated metabolic comorbidities being considered suitable candidates for the procedure [80, 81]. Based on current consensus, patients with severe esophagitis, severe bile reflux, or Barrett's metaplasia should be excluded by non-experts from consideration for OAGB, ensuring optimal outcomes and minimising complications until more evidence is available.

## Conclusion

This robust long-term data positions OAGB as a highly effective and safe primary operation and revisional procedure offering significant benefits for patients with obesity and related comorbidities. Overall, OAGB demonstrates favourable outcomes, making it an effective and safe option for both primary and revisional bariatric interventions.

## Supplementary Information

Below is the link to the electronic supplementary material.

Supplementary file1 (PPTX 6159 KB)

## Data Availability

The data that support the findings of this study are available from the corresponding author, [A.T.M], upon reasonable request.

## Declarations

### Permission to Reproduce Material from other Sources

No external materials other than what is already published was used in this article.
