# Supplementary figures and images for: Long-Term Outcomes of One Anastomosis Gastric Bypass: A Systematic Review and Meta-Analysis of 5-Year and Beyond
Source: Obes Surg. 2025 Oct 15;36(1):71–87. doi: 10.1007/s11695-025-08339-w (PMC12852249; doi:10.1007/s11695-025-08339-w)

## Slide 1
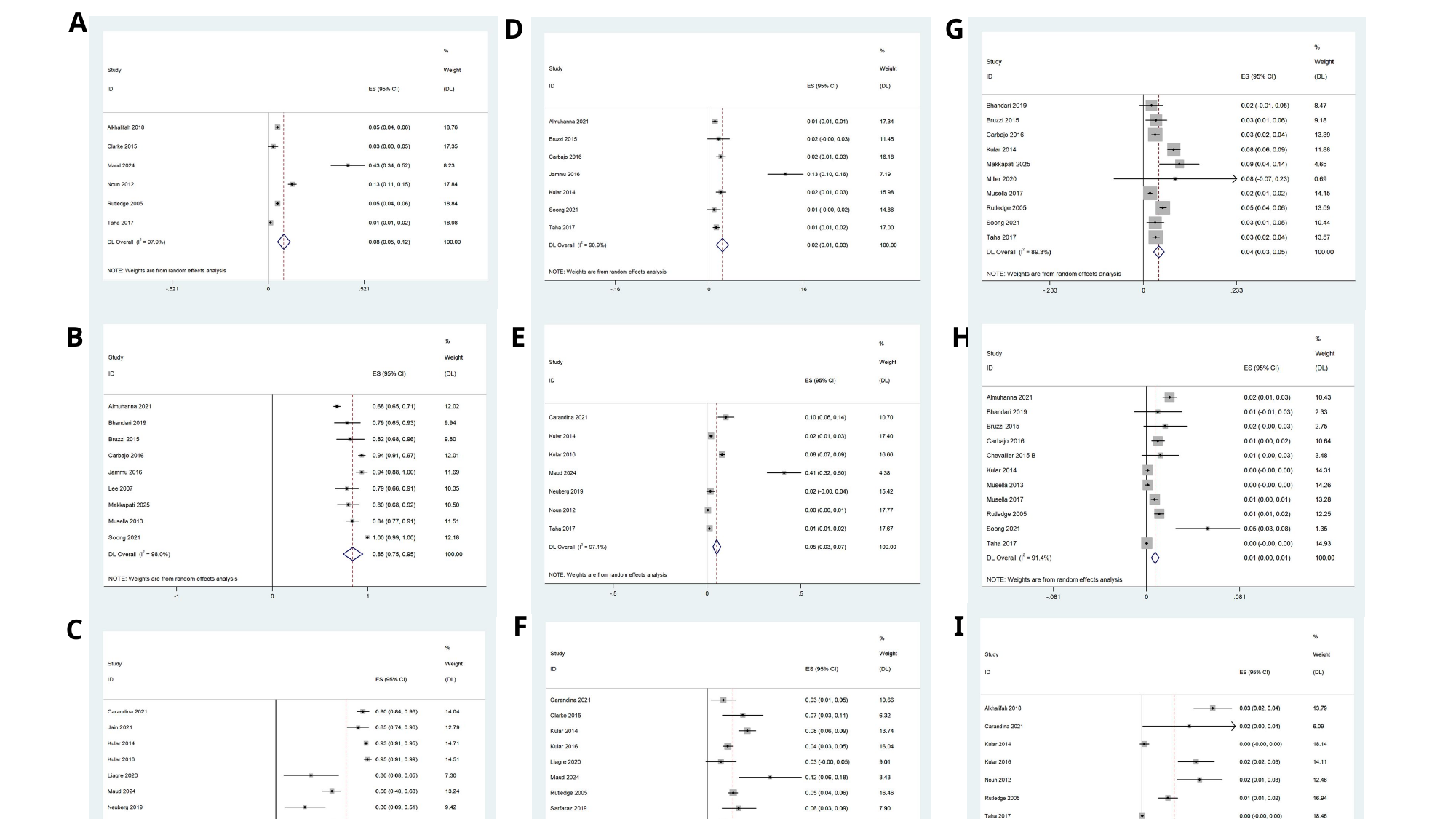

A
D
G
E
H
B
F
I
C

Supplement: Supplementary file 1 — Supplementary file1 (PPTX 6159 KB) [file 11695_2025_8339_MOESM1_ESM.pptx]
